# Audit of baseline cardiometabolic monitoring for patients prescribed or advised dose increase of antipsychotic medication by the knowsley assessment team

**DOI:** 10.1192/bjo.2021.479

**Published:** 2021-06-18

**Authors:** Debakanta Behera, Joanna Metcalf, Aaliya Adnan

**Affiliations:** 1 NWBH NHS Foundation Trust; 2 NWBH NHS FT

## Abstract

**Aims:**

To ascertain whether baseline monitoring of cardiometabolic health parameters was undertaken for patients prescribed dose increases of, antipsychotic medications in an outpatient setting. Whether results from baseline tests were taken into consideration when prescribing antipsychotic medications.

**Background:**

People with Severe Mental Illness have a reduction in life expectancy of 15-20 years. Chief factors implicated in this rate are smoking, obesity, metabolic dysfunction from diabetes, hypertension and stroke. Antipsychotic medications themselves are associated with increased risk of adverse cardiometabolic effects. The CATIE Study of patients prescribed atypical antipsychotics found that men were 85%, and women 137% more likely to have metabolic syndrome than control. Relative risk for type 2 diabetes and CHD in patients with metabolic syndrome is 1.5-5 times that of the general population.

**Method:**

The Team caseload was accessed between the 6/11/18-13/11/18. Chronologically the first 40 patients on the list who had been prescribed an antipsychotic or advised re a dose increase of antipsychotic chosen. Data were then retrospectively collected from informatics and progress notes, document uploads, initial assessments and the ICE bloods system to populate an excel spreadsheet which is currently in use within North West Boroughs.

**Result:**

Of the 40 patients, 50% (20) attended for physical health review. All who did not attend initial appointment were offered a second appointment. 15% (6) did not attend 2 appointments. 35% (14) were not offered a physical health appointment. 1 patient had BP documented (from full physical review during previous episode within 12 m). 2 patients had BMI documented; Smoking, alcohol and drug use status was documented in 42.5%(17), 57.5%(23) and 67.5%(27) of patients, respectively. And 67.5% (27) of patients had an HbA1c result within past 12 months on ICE and 62.5% (25) had lipid profile. At least 10 of these bloods were not requested by our team. 7 patients were given a blood form but did not have bloods done. 57% (4 of 7) abnormal HbA1c's were acknowledged and 20% (1 of 5) lipid profiles.

**Conclusion:**

This audit demonstrates that baseline cardiometabolic monitoring could be improved for patients under the Assessment Team who are prescribed antipsychotics. Only half of the audited patients had had a physical health review, despite being prescribed, or their GP being advised regarding an increase in dose of, antipsychotic medication. It is important to note that 15% of patients were offered but failed to attend an appointment for physical health review.

